# Supplementary material for: Calculating Protein Content of Expressed Breast Milk to Optimize Protein Supplementation in Very Low Birth Weight Infants with Minimal Effort—A Secondary Analysis
Source: Nutrients. 2020 Apr 27;12(5):1231. doi: 10.3390/nu12051231 (PMC7282015; doi:10.3390/nu12051231)
Supplement: Supplementary file 1 [file nutrients-12-01231-s001.zip › Figure S1.pdf]

Pat$_{adj}$ 1

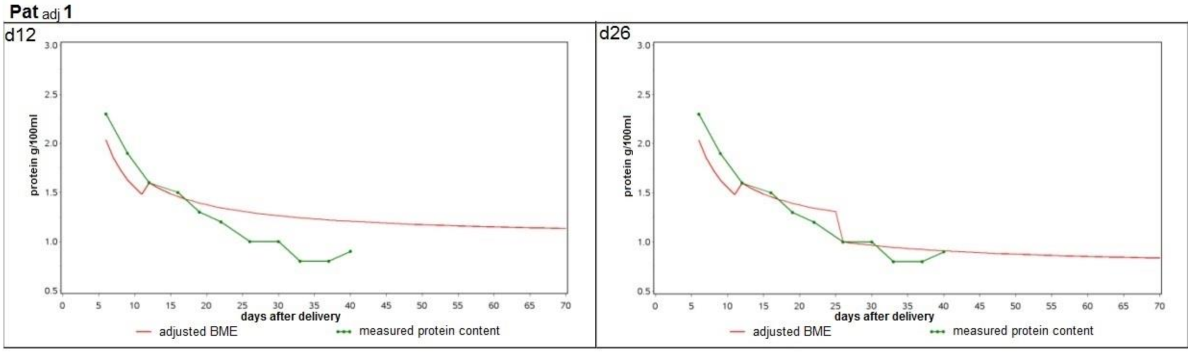

Pat$_{adj}$ 2

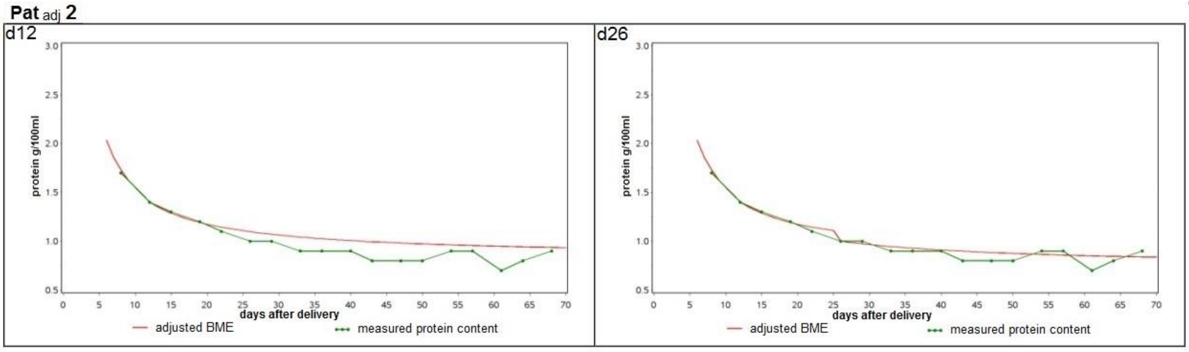

Pat$_{adj}$ 3

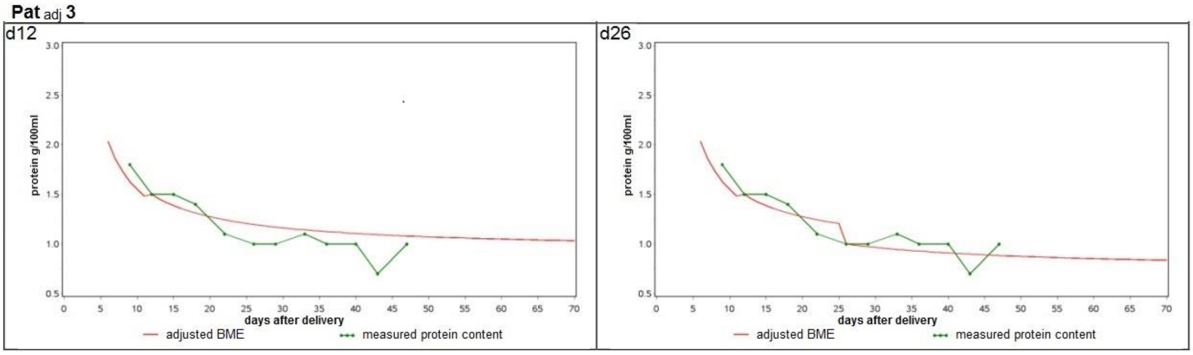

Pat$_{adj}$ 4

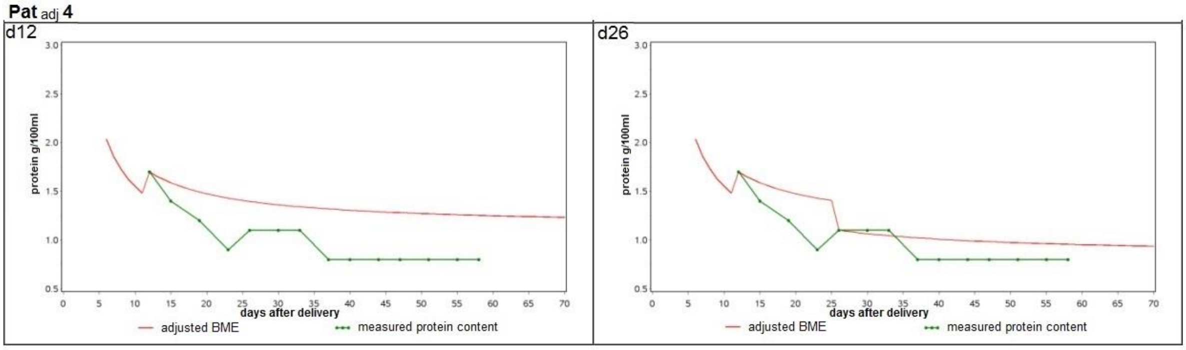

Pat$_{adj}$ 5

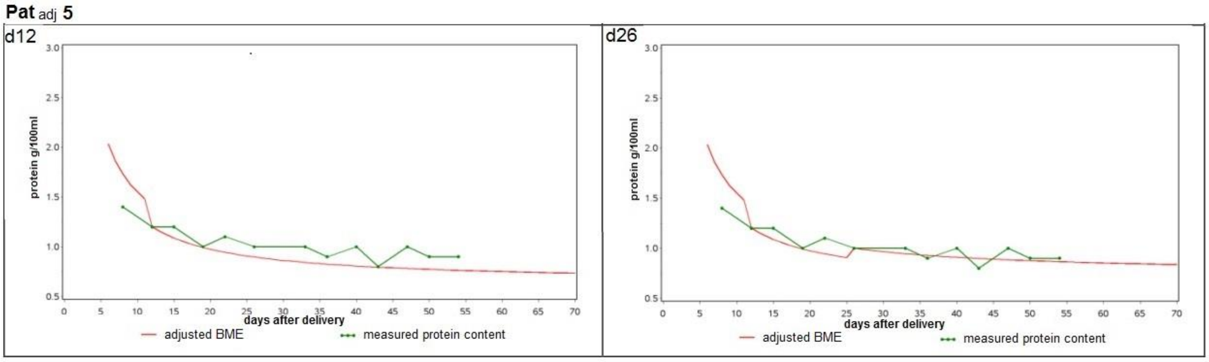

Pat $_{adj}$ **6**

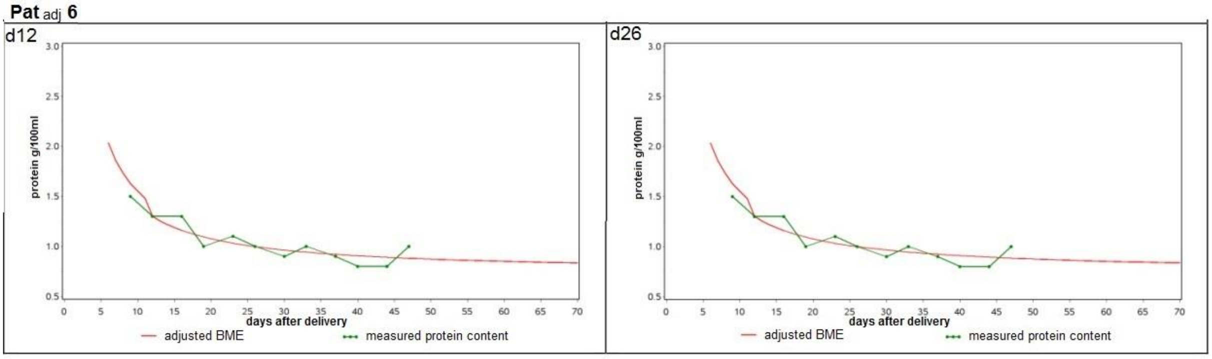

Pat $_{adj}$ **7**

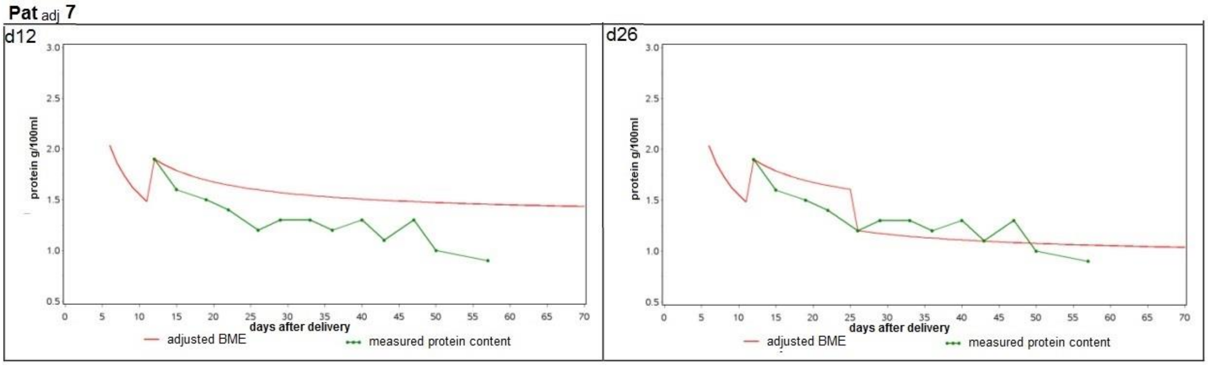

Pat $_{adj}$ **8**

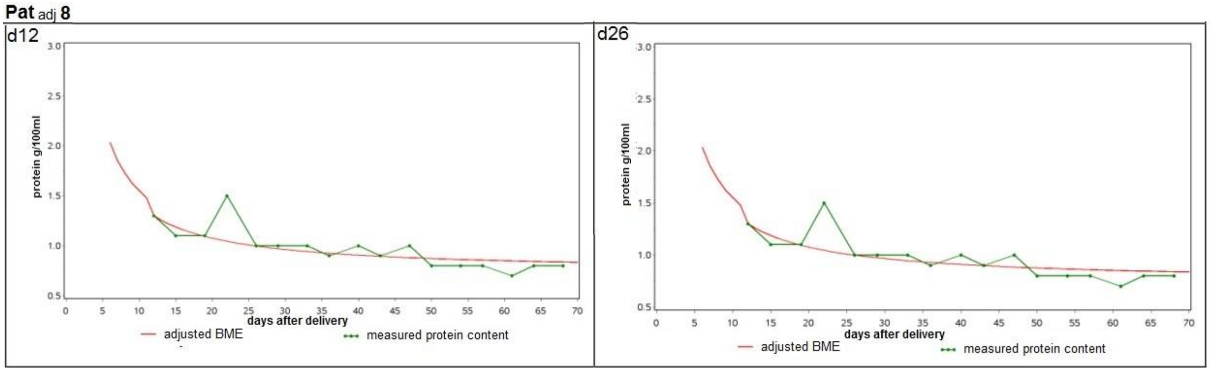

Pat $_{adj}$ **9**

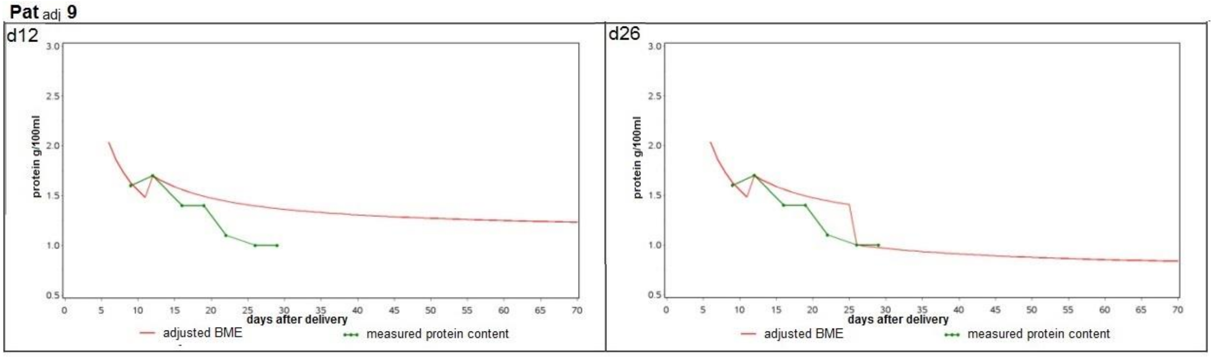

Pat $_{adj}$ **10**

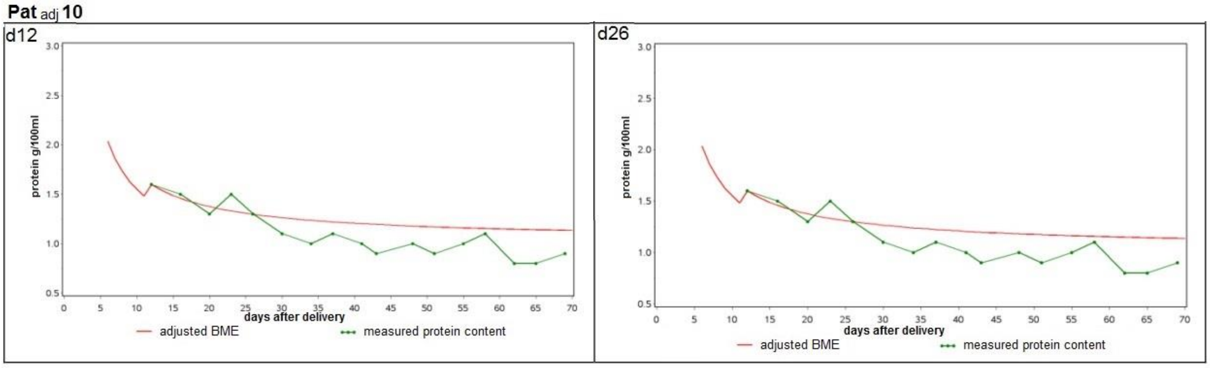

Figure S1: Adjustment of the 'breast milk-equation (BME)' of the validation cohort (10 patients; Pat_{adj}1-10) on day 12 and 26 after delivery by measured actual protein content
