# Supplementary material for: Calculating Protein Content of Expressed Breast Milk to Optimize Protein Supplementation in Very Low Birth Weight Infants with Minimal Effort—A Secondary Analysis
Source: Nutrients. 2020 Apr 27;12(5):1231. doi: 10.3390/nu12051231 (PMC7282015; doi:10.3390/nu12051231)
Supplement: Supplementary file 1 [file nutrients-12-01231-s001.zip › Figure S2.pdf]

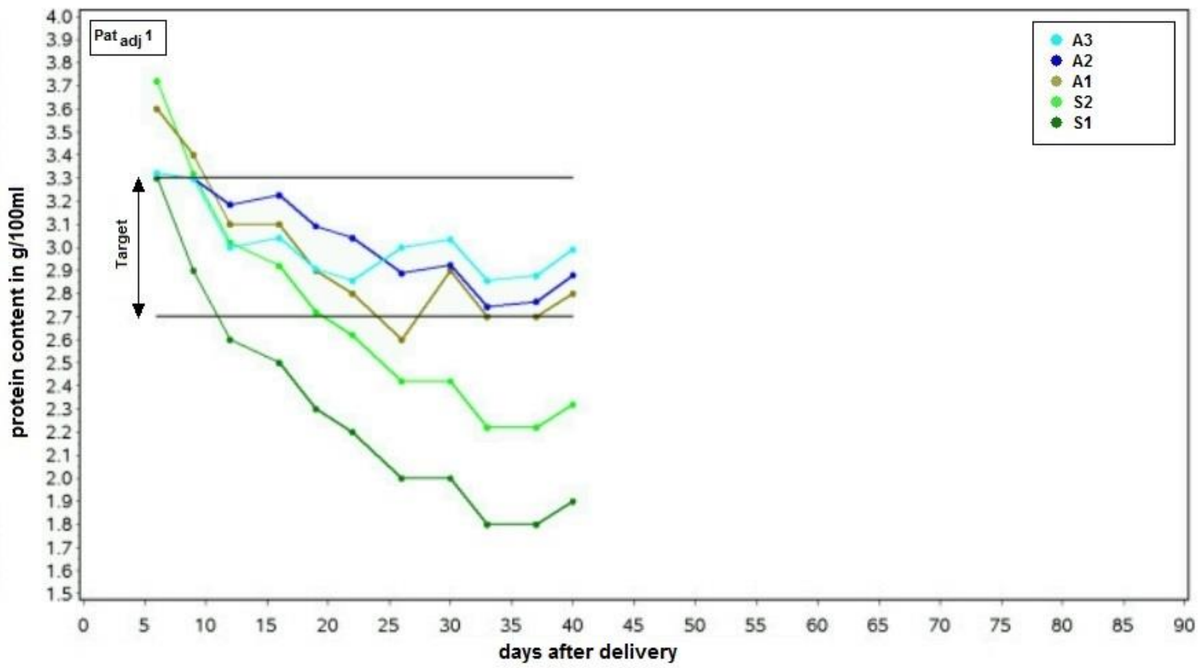
Pat adj 1
A3
A2
A1
S2
S1
Target
protein content in g/100ml
days after delivery

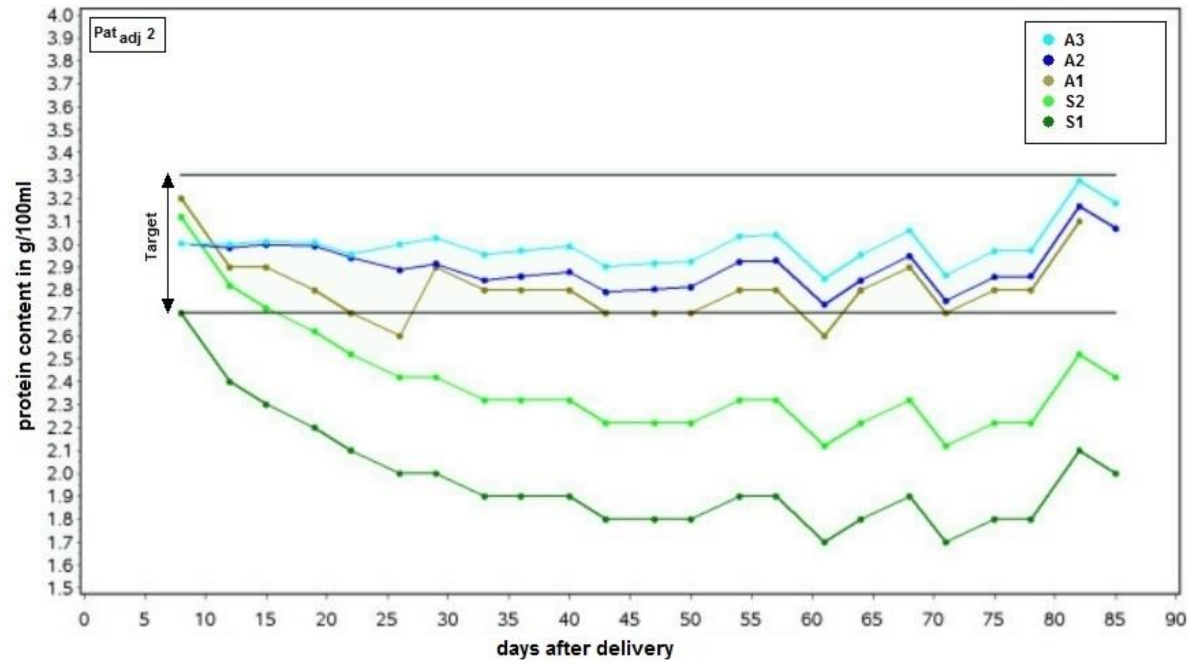
Pat adj 2
A3
A2
A1
S2
S1
Target
protein content in g/100ml
days after delivery

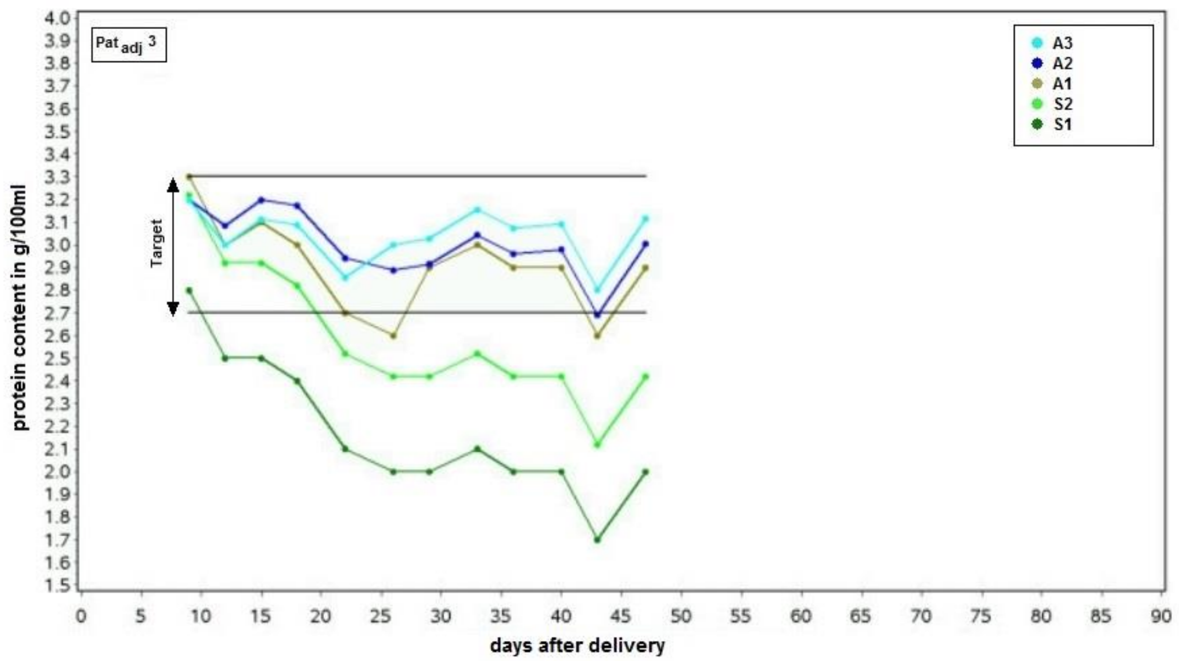
Pat adj 3
A3
A2
A1
S2
S1
Target
protein content in g/100ml
days after delivery

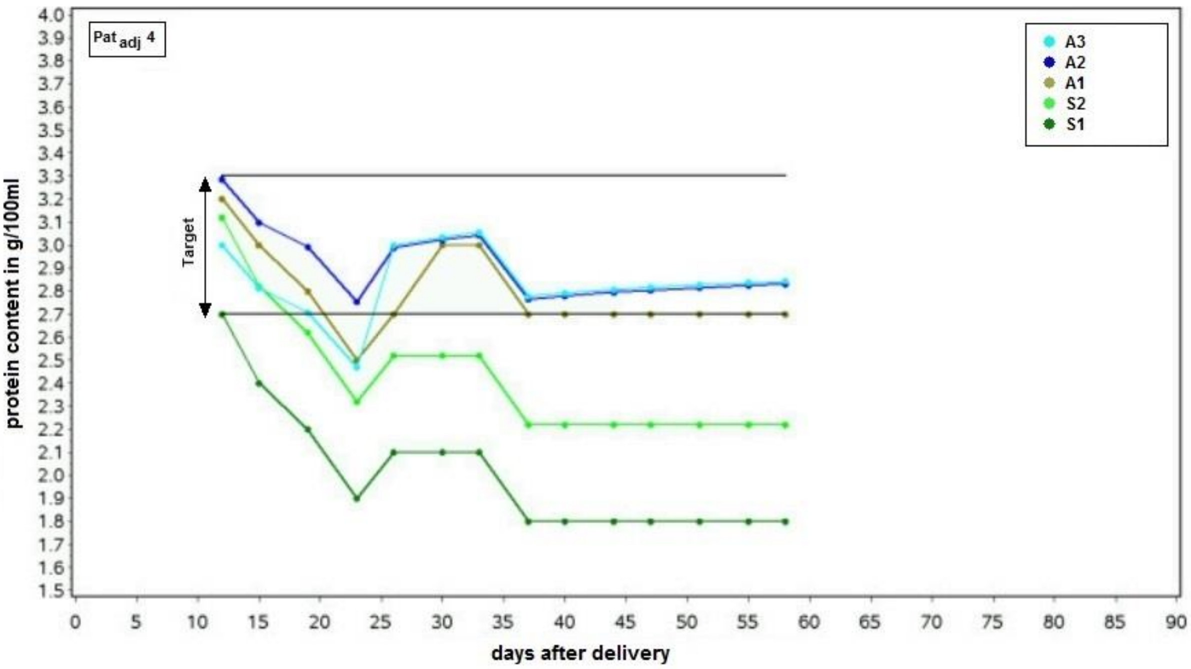
Pat adj 4
A3
A2
A1
S2
S1
Target
protein content in g/100ml
days after delivery

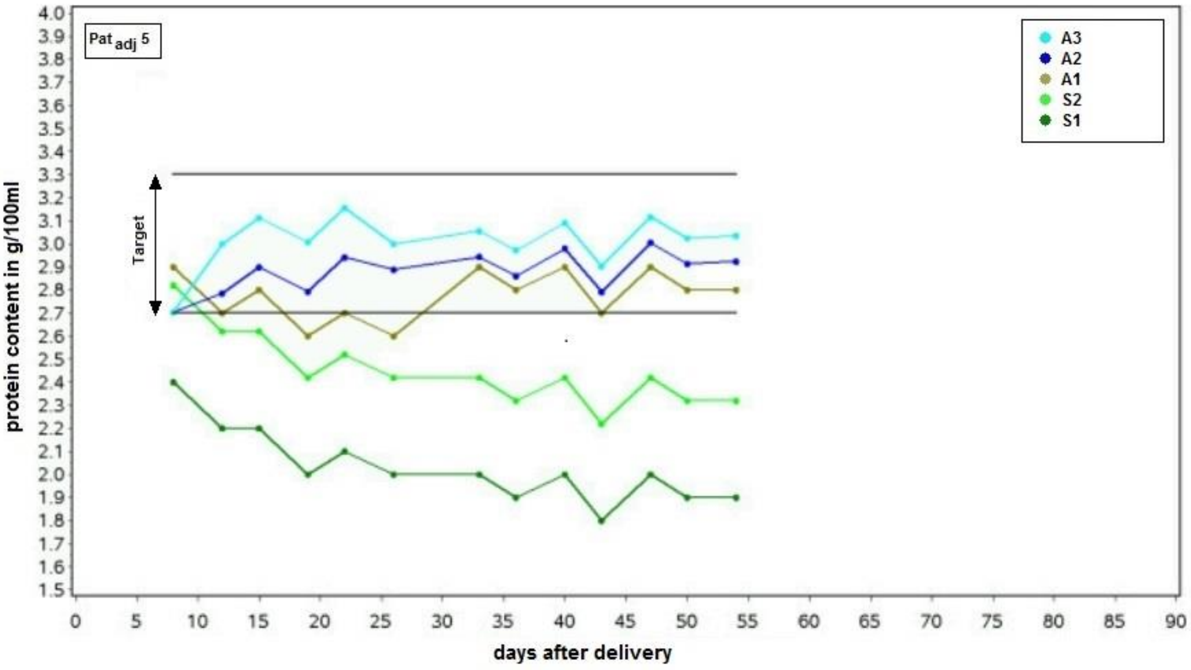
Pat adj 5
A3
A2
A1
S2
S1
Target
protein content in g/100ml
days after delivery

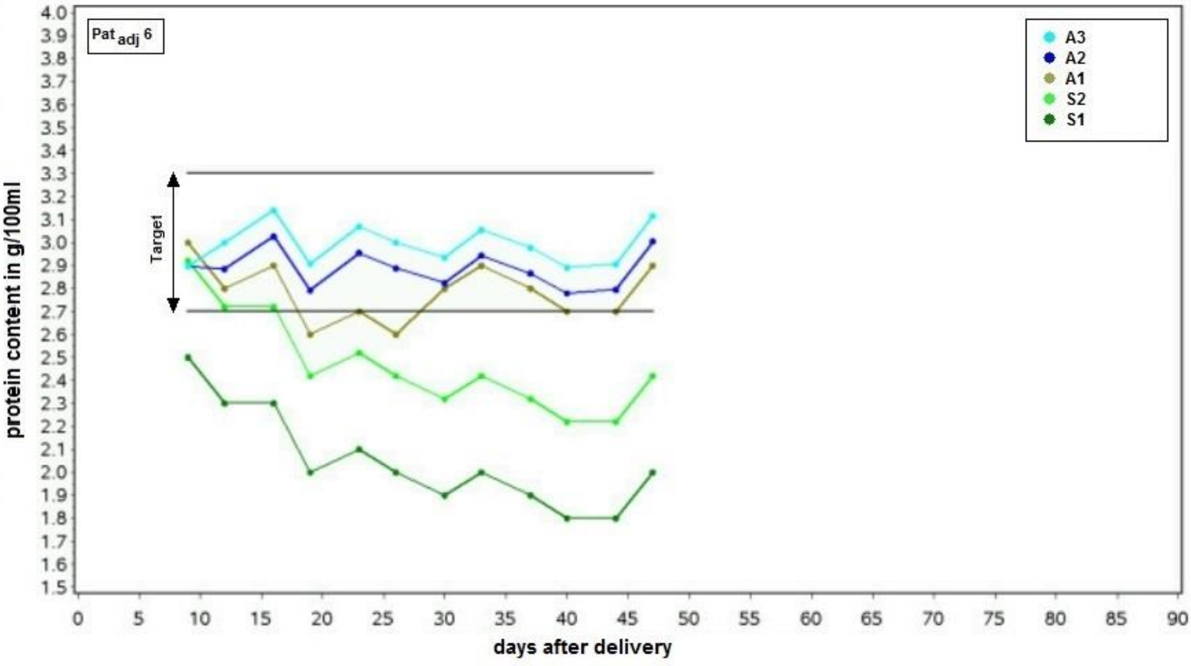
Pat adj 6
A3
A2
A1
S2
S1
Target
protein content in g/100ml
days after delivery

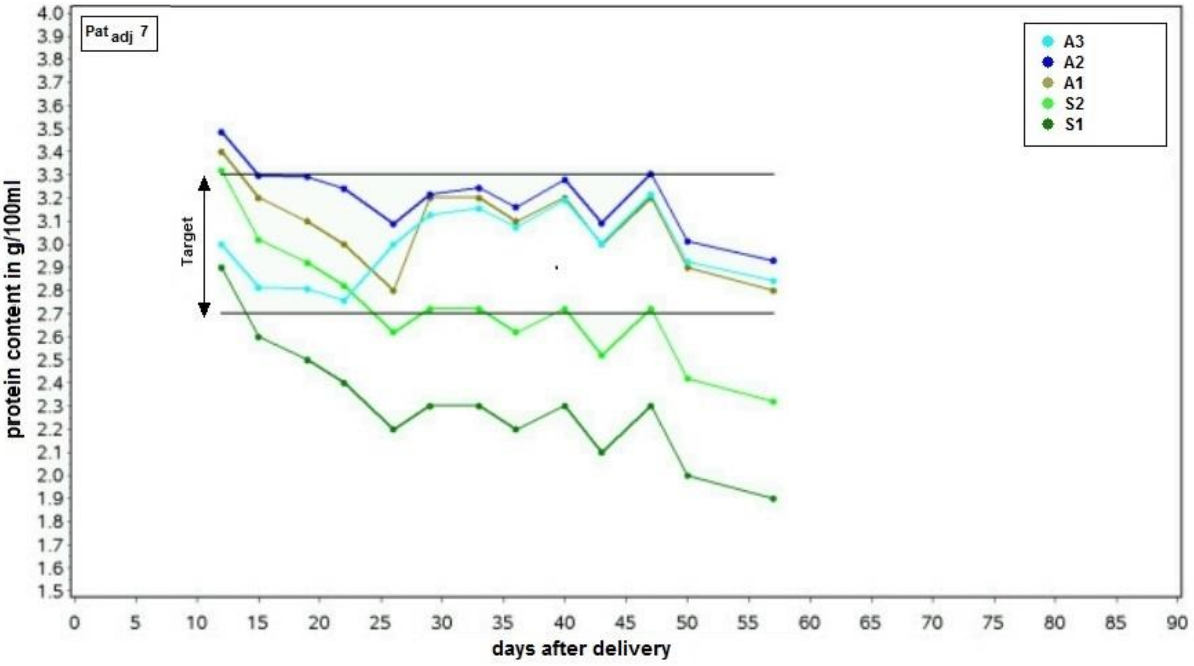
Pat adj 7
A3
A2
A1
S2
S1
Target
protein content in g/100ml
days after delivery

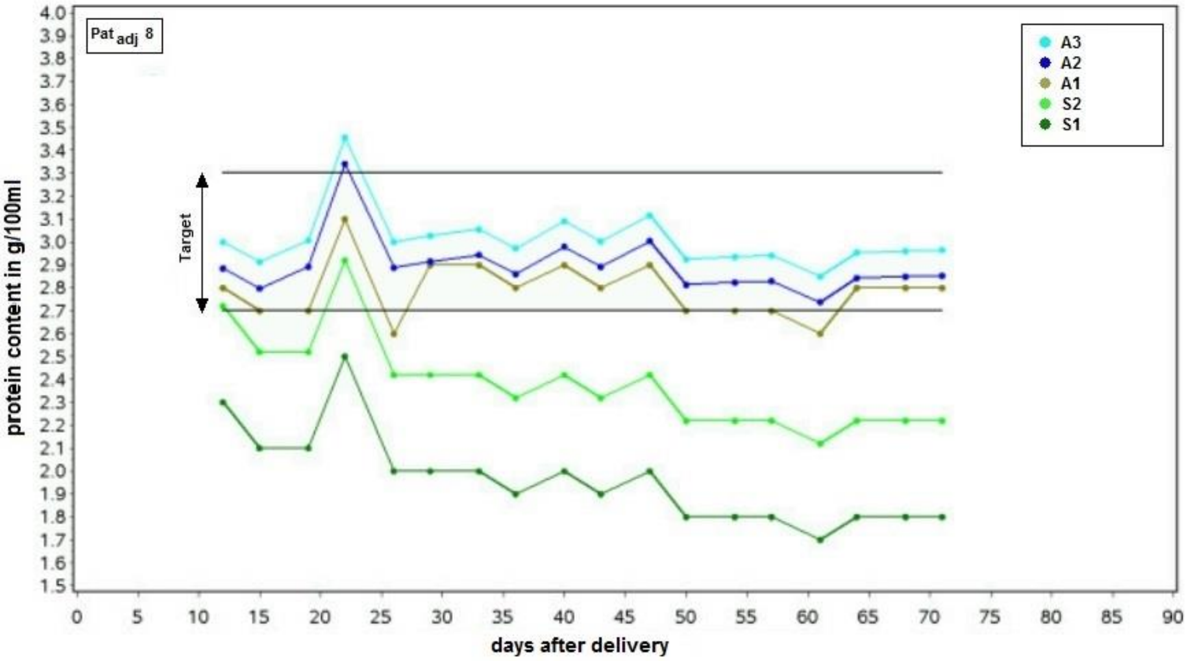
Pat adj 8
A3
A2
A1
S2
S1
Target
protein content in g/100ml
days after delivery

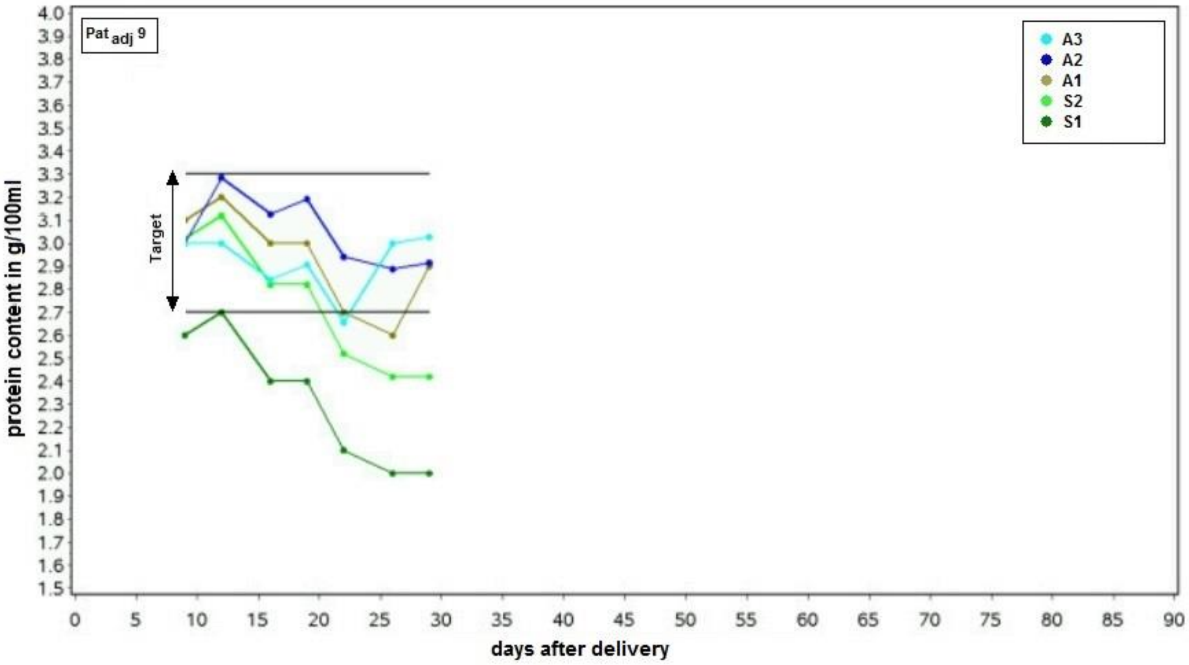
Pat adj 9
A3
A2
A1
S2
S1
Target
protein content in g/100ml
days after delivery

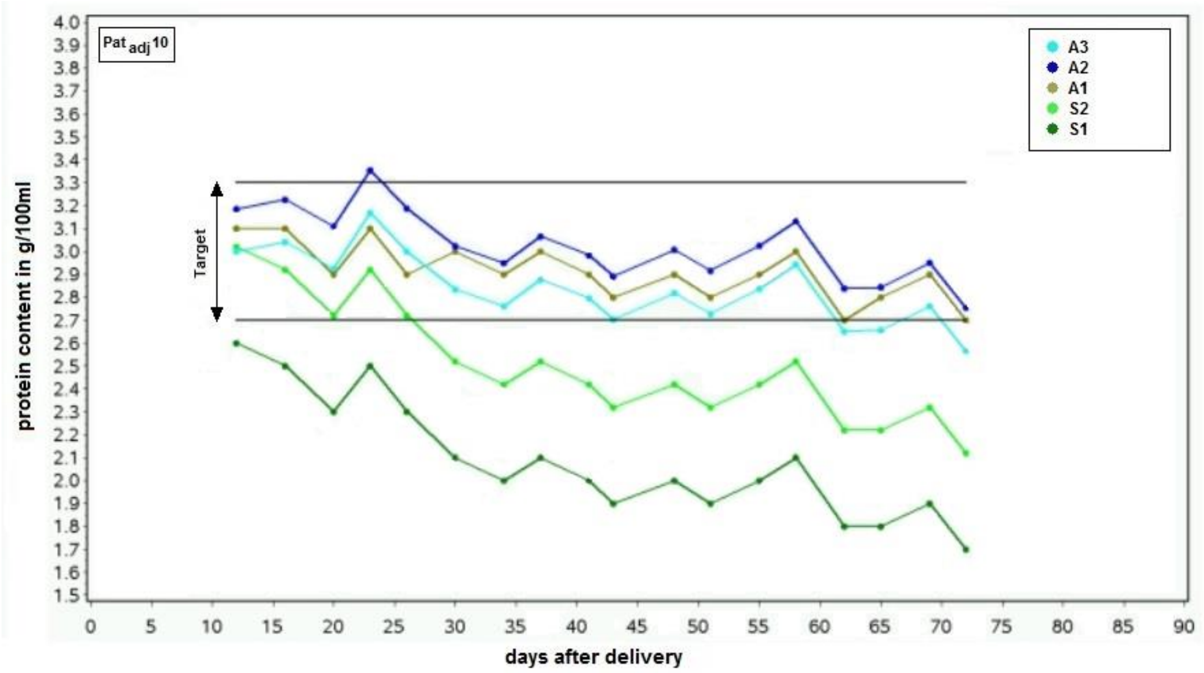

Figure S2: Comparing the resulting protein supply performing the 5 different supplementations strategies (S1: standardized supplementation adding 1g protein/100ml breast milk; S2: standardized supplementation adding 1.42g protein/100ml breast milk; A1: adding protein according to protein content of preterm breast milk after Fenton et al. [13]; A2: adding protein according to calculated breast milks' protein content by using the 'breast milk-equation (BME)'; A3: adding protein according to calculated breast milks' protein content by using the 'breast milk-equation and adjusting twice on day 12 and 26 by measured actual protein content) in the validation cohort (10 patients; Pat_{adj}1-10)
